# Bone Marrow Endothelial Cells Influence Function and Phenotype of Hematopoietic Stem and Progenitor Cells after Mixed Neutron/Gamma Radiation

**DOI:** 10.3390/ijms20071795

**Published:** 2019-04-11

**Authors:** Lynnette Cary, Daniel Noutai, Rudolph Salber, Opeyemi Fadiyimu, Arthur Gross, Graca Almeida-Porada, Yared Kidane, Mark Whitnall

**Affiliations:** 1 Scientific Research Department, Armed Forces Radiobiology Research Institute, Bethesda, MD 20889, USA; 2 Walter Reed National Naval Medical Center, Bethesda, MD 20889, USA; daniel.noutai.mil@mail.mil; 3 Department of Clinical Studies, University of Pennsylvania, Philadelphia, PA 19104, USA; salber@vet.upenn.edu; 4 Navy Medicine Training Support Center, Fort Sam Houston, TX 78234, USA; opeyemi.fadiyimu.mil@mail.mil; 5 Department of Osteopathic Medicine, Liberty University, Lynchburg, VA 24502, USA; Asgross@liberty.edu; 6 Institute for Regenerative Medicine, Wake Forest University, Winston-Salem, NC 27101, USA; galmeida@wakehealth.edu; 7 KBRwyle, NASA Johnson Space Center, Houston, TX 77058, USA; yared.h.kidane@nasa.gov; 8 Scientific Research Department, Armed Forces Radiobiology Research Institute, Bethesda, MD 20889, USA; markhwhitnall@gmail.com

## Abstract

The bone marrow (BM) microenvironment plays a crucial role in the maintenance and regeneration of hematopoietic stem (HSC) and progenitor cells (HSPC). In particular, the vascular niche is responsible for regulating HSC maintenance, differentiation, and migration of cells in and out of the BM. Damage to this niche upon exposure to ionizing radiation, whether accidental or as a result of therapy, can contribute to delays in HSC recovery and/or function. The ability of BM derived-endothelial cells (BMEC) to alter and/or protect HSPC after exposure to ionizing radiation was investigated. Our data show that exposure of BMEC to ionizing radiation resulted in alterations in Akt signaling, increased expression of PARP-1, IL6, and MCP-1, and decreased expression of MMP1 and MMP9. In addition, global analysis of gene expression of HSC and BMEC in response to mixed neutron/gamma field (MF) radiation identified 60 genes whose expression was altered after radiation in both cell types, suggesting that a subset of genes is commonly affected by this type of radiation. Focused gene analysis by RT-PCR revealed two categories of BMEC alterations: (a) a subset of genes whose expression was altered in response to radiation, with no additional effect observed during coculture with HSPC, and (b) a subset of genes upregulated in response to radiation, and altered when cocultured with HSPC. Coculture of BMEC with CD34+ HSPC induced HSPC proliferation, and improved BM function after MF radiation. Nonirradiated HSPC exhibited reduced CD34 expression over time, but when irradiated, they maintained higher CD34 expression. Nonirradiated HSPC cocultured with nonirradiated BMEC expressed lower levels of CD34 expression compared to nonirradiated alone. These data characterize the role of each cell type in response to MF radiation and demonstrate the interdependence of each cell’s response to ionizing radiation. The identified genes modulated by radiation and coculture provide guidance for future experiments to test hypotheses concerning specific factors mediating the beneficial effects of BMEC on HSPC. This information will prove useful in the search for medical countermeasures to radiation-induced hematopoietic injury.

## 1. Background

The need to maintain a secure environment in the current nuclear climate is ever relevant. Terrorism involving improvised nuclear devices (IND), radiation exposure devices, radiation dispersal devices, or an attack on a nuclear power plant or a facility/vehicle that houses radioactive materials may lead to devastating injury or death to few or many due to exposure to low or high linear energy transfer (LET) radiation. Observations from personnel exposed to high levels of mixed neutron/gamma radiation, here referred to as Mixed Field (MF), not only show the severity of the acute radiation syndrome (ARS), but also demonstrate that treatment requirements are complex. This has prompted extensive detailed studies on the pathophysiology of various systems due to radiation injury, including hematopoietic organs. Because rapidly replicating cells, including hematopoietic cells, are most sensitive to the acute effects of ionizing radiation (IR), there is ongoing effort to protect the hematopoietic system from damage. Early work demonstrated that shielding the spleen from radiation, as well as injecting splenocytes or bone marrow (BM) cells provided radiation protection [1,2].

BM cells, while susceptible to IR, maintain a radiation resistant subpopulation of cells [3,4] and BM reconstitution remains a therapeutic approach to ARS reviewed in [5]. Stem cell (SC) transplantation and platelet transfusions, as critical components to recovery, have been demonstrated both by clinical and basic research. Other cell types, including stromal cells, induced pluripotent, and mesenchymal SC, are under investigation as sources of cells to reconstitute radiation damaged bone marrow [6].

Mobilization of hematopoietic stem/hematopoietic stem and progenitor cells (HSC/HSPC) from the BM into circulation has immense clinical relevance, including the well-established treatment for malignancies such as multiple myeloma (MM), lymphomas, and leukemias [7,8]. HSC transplantation has become a standard of care after radiation, both in a therapeutic setting and in the event of accidental radiation exposure [9]. However, this treatment is not without complications, including infection, pulmonary, cardiac, and endocrine effects. Understanding the environment in which these cells exist, alterations in the environment and to HSPC due to stress/injury, as well as indications of how to modulate function including mobilization, are key to the optimal use of these cells with minimal negative effects. Levels of GCSF can be increased by radiation [10] and may affect mobilization and maturation of SC [11,12], and agents that increase GCSF levels have proven to be potent radiation countermeasures [13,14]. GCSF (both Neupogen^R^ and Neulasta^R^) are FDA-approved for the treatment of ARS [15]. 

HSC function in both normal and pathophysiological conditions is an area of active research; this includes HSC capability for both self-renewal and development into cells of multiple hematopoietic lineages. HSPC have been detected in endosteal and vascular environments [16,17,18], and there has been much effort in understanding their development, differentiation, and function within those environments. Work utilizing bone morphogenetic protein receptor mutant mice correlated an increase in osteoblasts with an increase in HSC, and further study showed HSC residing near and binding to osteoblasts lining the bone marrow surface through adhesion molecule interactions [19,20]. However, other studies demonstrated that HSPC do not express N-cadherin necessary for adhesion to osteoblasts [21]. A decrease in osteoblasts did not correlate with a decrease in HSC. Analysis of localization of HSCs in the bone marrow using SLAM family receptors revealed that most HSC reside on the surface of sinusoidal blood vessels [22], suggesting that HSC might be maintained in a perivascular niche by endothelial or perivascular cells [23,24]. Multiple cell types and cytokines facilitate the maintenance of the SC niche, including mesenchymal progenitor cells, CXCL12 [25], stem cell factor (SCF), and endothelial cells (EC). The SC environment and signaling are modulated by injury. Real-time imaging revealed that HSC injected intravenously migrate to the bone marrow within hours, and the specific homing location within marrow varies in irradiated versus nonirradiated mice [17].

The SC effect involves more than simply improved mobilization. Other cell types or factors may be required for optimal function, and EC represent one such cell type. Irradiated HSC were capable of recovery and expansion in the presence of human EC [26]. BMEC secrete proteins such as pleiotrophin and epidermal growth factor, both which are vital for hematopoietic regeneration after total-body irradiation (TBI) [27,28]. With the knowledge of EC contribution to SC recovery from injury established, further studies on mechanisms of SC mobilization and maturation during radiation injury, and the role of EC in this process, is critical in designing beneficial strategies to overcome radiation damage to the hematopoietic system. 

With this in mind, studies were performed to determine the effect(s) of MF radiation on EC and CD34+ HSPC cultured independently. These were compared to cocultures of EC and HSPC in a radiation model where neither cell type, both cell types, or one cell type was irradiated and cultured with the alternate cell type. The use of MF radiation, incorporating a high LET neutron component, increased the radiation challenge. Our data show that radiation induced Akt signaling in EC. BMEC also responded to MF by increasing expression of PARP-1 and secretion of IL6. Both proteins contribute to a proinflammatory profile in response to MF radiation. Metalloproteinase expression was altered by MF radiation and EC specific genes were upregulated in response to MF. Coculture of BMEC with nonirradiated HSPC enhanced proliferation of HSPC, but coculture had no effect on proliferation of irradiated HSPC. Coculture slightly enhanced colony formation in irradiated HSPC, suggesting an effect on survival and differentiation. This was supported by flow cytometry data using CD34 expression. Coculture of BMEC with HSPC after MF radiation modified CD34+ surface marker expression on HSPC, likely an indication of maturation. In addition to analyzing the effects of BMEC on HSPC, we investigated the effect of HSPC on BMEC. HSPC had no effect on expression of EC specific genes, but attenuated the radiation-induced growth factor expression in EC, as well as phospholipase A2 expression. Increased phospholipase A2 is correlated with EC survival after radiation via Akt and MAPK signaling [29], and the observation of reduced phospholipase A2 in the presence of HSPC suggests that HSPC may affect EC cell viability. While much focus is on preserving an adequate number of HSPC to repopulate the immune system after IR, EC preservation is also a relevant strategy. These data advance our understanding of cell support and/or communication during injury, and support our hypothesis that the identification of therapeutics targeting EC survival is a valid approach.

## 2. Statistical Analysis

Data were analyzed for statistical significance using *t*-test or ANOVA with Dunnett post-test. Significance was set at *p* < 0.05 (*) and *p* < 0.005 (**). Experiments were performed two or three times and each sample was in triplicate except microarray profiling. Two independent microarray experiments were performed and data shown represent one experiment.

## 3. Results

### 3.1. MF Radiation-Induced Akt Signaling in hBMEC.

**Figure 1:**
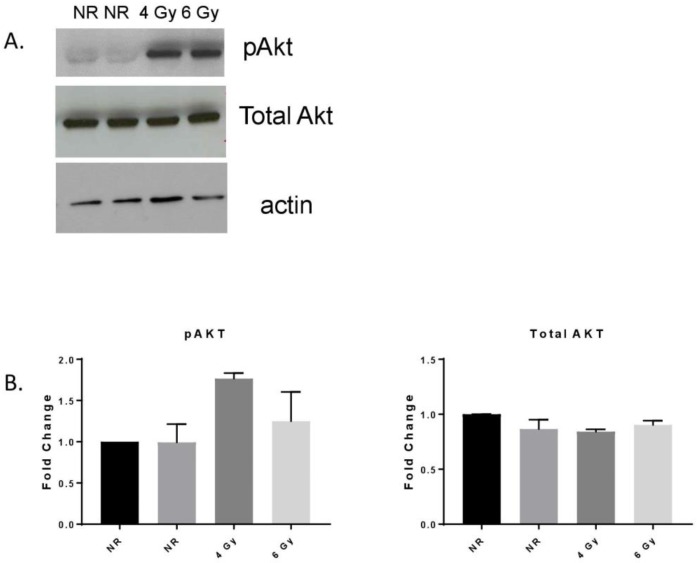
MF-radiation exposure activated Akt signaling in hBMEC. Cells were nonirradiated (NR) (2 samples) or irradiated (R) with 4 or 6 Gy. Cells were lysed 4 h after exposure and Akt phosphorylation was detected by Western blot (**A**). Densitometry was performed on three independent experiments, normalized to actin (**B**). Error bars represent standard deviation. The pAKT level in the 4 Gy sample was near significance (*p* = 0.0501) compared to NR.

Phosphorylation of the serine threonine protein kinase Akt stimulates catalytic activity, resulting in the phosphorylation of proteins that affect cell growth, cell cycle entry, and cell survival. EC signaling after ionizing radiation involves Akt activation [30]. MF radiation (4 Gy, 6 Gy) of hBMEC induced Akt phosphorylation 4 h after exposure without changes in total Akt protein expression (Figure 1). 

### 3.2. MF Radiation Altered Protein Expression in hBMEC.

**Figure 2:**
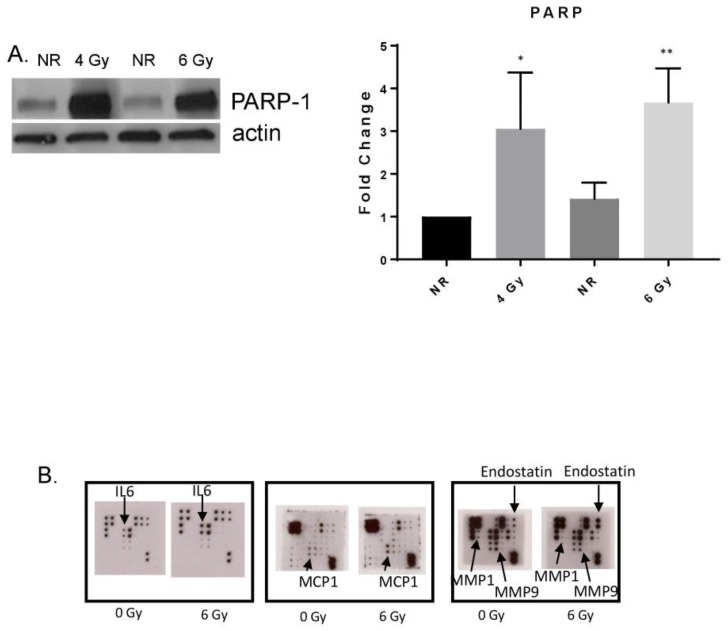
MF radiation increased protein expression in BMEC. (**A**) Increased PARP-1 expression was detected 4 h postradiation (6 Gy). Densitometry was performed on three independent experiments, normalized to actin. * *p* = 0.0326; ** *p* = 0.0086. Significance was determined compared to NR conditions. Error bars represent standard deviation. (**B**) Secreted proteins were analyzed from cell culture supernatant 24 h post-radiation (6 Gy) using antibody arrays.

PARP-1 expression was also increased after 6 Gy MF radiation (Figure 2A). PARP-1 is involved in both DNA repair and the pathogenesis of inflammation. Alterations in expression of proteins known to be modulated in response to gamma radiation were observed after MF radiation exposure [23,31,32,33]. Protein arrays revealed increases in the cytokines IL6, monocyte-chemoattractant protein-1 (MCP-1), and endostatin, and decreases in matrix metalloproteinases (MMP) −1 and −9 (Figure 2B). 

### 3.3. MF Radiation and CD34+ Coculture Altered mRNA Expression in hBMEC

**Figure 3:**
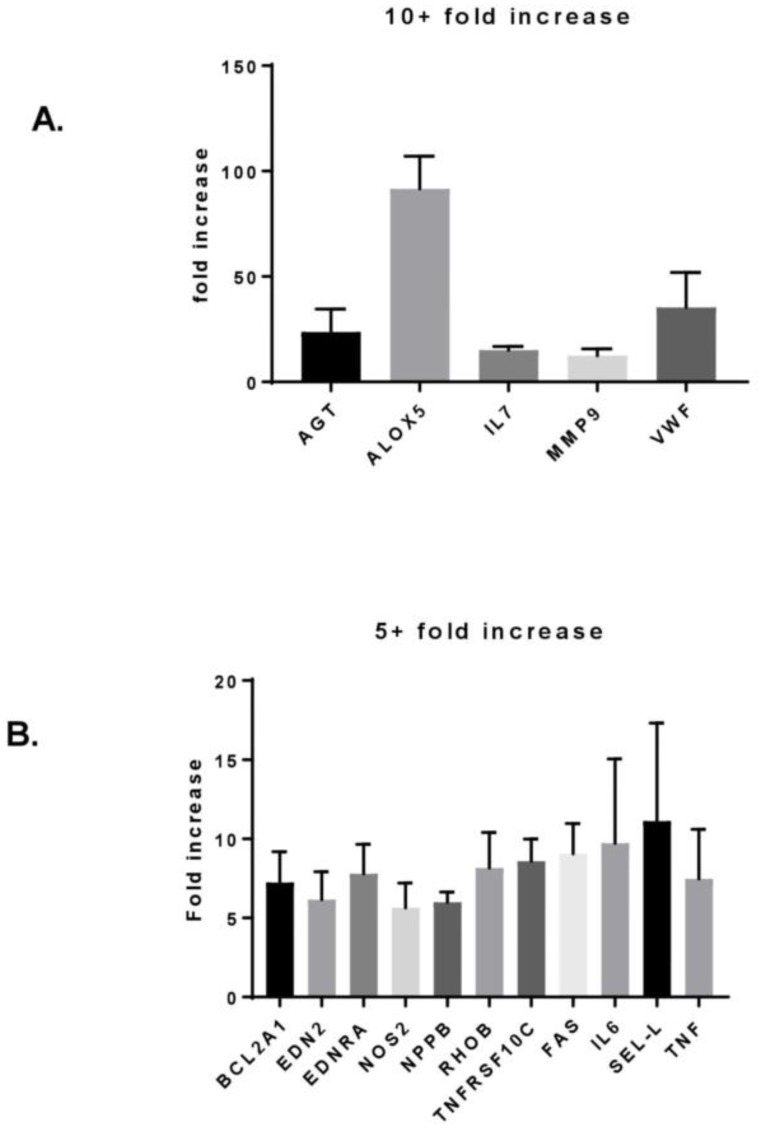
MF radiation induced changes in hBMEC gene expression. Cells were irradiated with 6 Gy MF. Four h after exposure, RNA was isolated and changes in gene expression were analyzed by RT-PCR array. Changes depicted were compared to nonirradiated hBMEC control cells. Error bars represent standard deviation. (**A**) Increased gene expression 10-fold or greater. (**B**) Increased gene expression 5-fold or greater.

Exposure to 6 Gy MF radiation increased expression of multiple genes, five of which were upregulated 10-fold or more (Figure 3A). AGT, VWF, and ALOX5 genes (encoding angiotensinogen, Von Willebrand Factor, and Arichidonate-5-lipoxygenase, respectively) are proteins specifically located in or involved with EC-mediated functions. MMP-9 message increased 12-fold compared to nonirradiated hBMEC, which is in contrast to the decrease in protein seen by protein array. The cytokine with the greatest increase in mRNA expression was IL7. 

**Figure 4:**
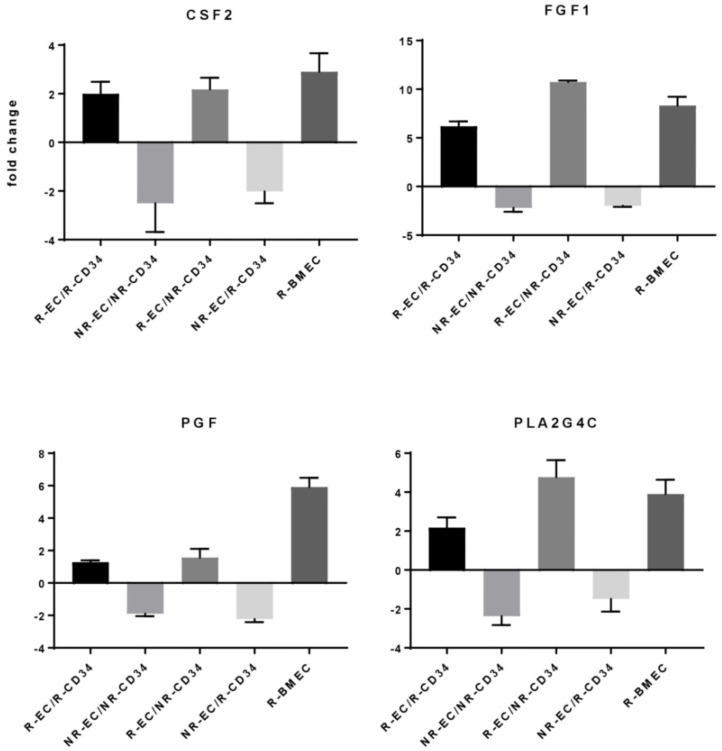
HSPC altered hBMEC gene response to MF radiation. Cells were irradiated with 6 Gy MF. HSPC were added to hBMEC and cocultured for 4 h. HSPC were removed, and RNA was isolated from hBMEC. Changes in gene expression were determined by RT-PCR array. Error bars represent standard deviation.

Moderate increases in 13 gene products (> 5 and < 10-fold) were detected (Figure 3B and Figure 4). Among these were IL6, a cytokine whose increase was also detected at the protein level. Most genes upregulated in response to radiation in hBMEC were not affected by the presence of CD34+ HSPC (Figure 3B). However, there were four genes (encoding GM-CSF, FGF1, PGF, and Phospholipase A2) whose expression pattern was upregulated in response to radiation and downregulated in response to coculture with CD34+ HSPC compared to nonirradiated hBMEC (Figure 4).

**Figure 5:**
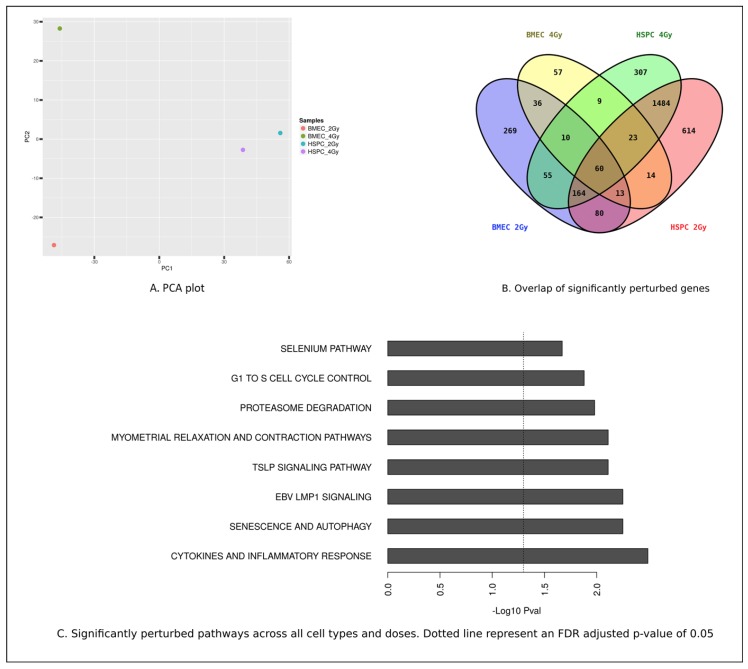
Bioinformatics analysis of HSPC and hBMEC in response to 2 Gy or 4 Gy MF radiation. (**A**) Principal component analysis plot (**B**) Overlap of significantly modulated genes, (**C**) Significantly perturbed pathways.

Global gene expression arrays were performed to analyze the general radiation induced response of both hBMEC and CD34+ HSPC. Our data confirm the radiosensitivity of HSPC. MF radiation exposure to CD34+ cells, at either 2 Gy or 4 Gy, resulted in alterations in expression level of 2112 or 2452 genes respectively. In contrast, MF exposure of hBMEC to either 2 Gy or 4 Gy resulted in alterations in expression level of 764 and 222 genes, respectively (Supplement Table S1). Principle component analysis revealed that variations in gene expression were greater between cell types than between radiation doses (Figure 5A). There are 60 genes whose expression levels are altered in all samples (Figure 5B). The top eight significantly modulated biological processes are shown in Figure 5C.

### 3.4. hBMEC Altered Stem Cell Proliferation

**Figure 6:**
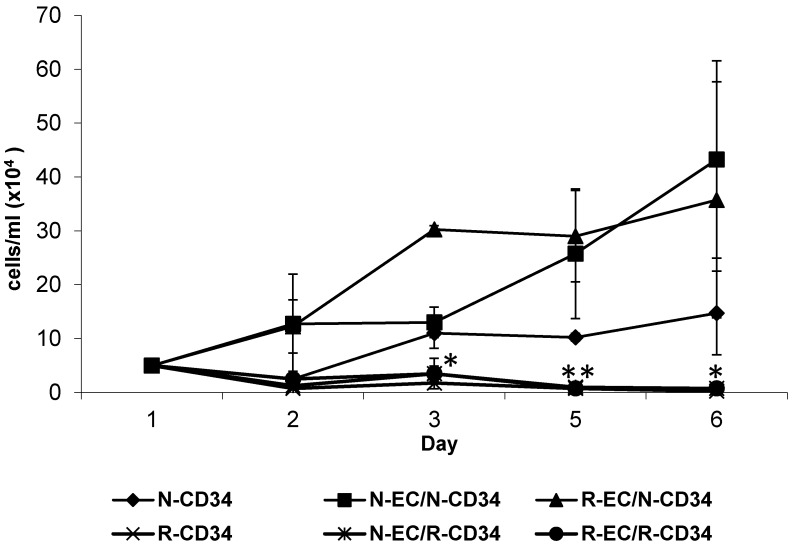
hBMEC improved HSPC proliferation. CD34+ HSPC or EC were irradiated (4 Gy MF) and cocultured overnight. N = nonirradiated; R = irradiated. CD34+ HSPC were counted using trypan blue exclusion. Significance was determined with samples compared to N-CD34+ cells. Error bars represent standard deviation. * *p* < 0.05. ** *p* <0.005.

SC renewal involves both differentiation and proliferation. One strategy for self-renewal involves asymmetric cell division, in which each stem cell divides to produce one daughter with a stem-cell fate (self-renewal) and one daughter that differentiates [34]. CD34+ HSPC show limited proliferation in vitro, true to the quiescence of SC. However, the presence of hBMEC, either nonirradiated or irradiated, stimulated proliferation of HSPC. Radiation of HSPC inhibited proliferation, and this effect was not rescued by the presence of hBMEC (Figure 6). 

### 3.5. hBMEC Improved HSPC Differentiation after MF Radiation

**Figure 7:**
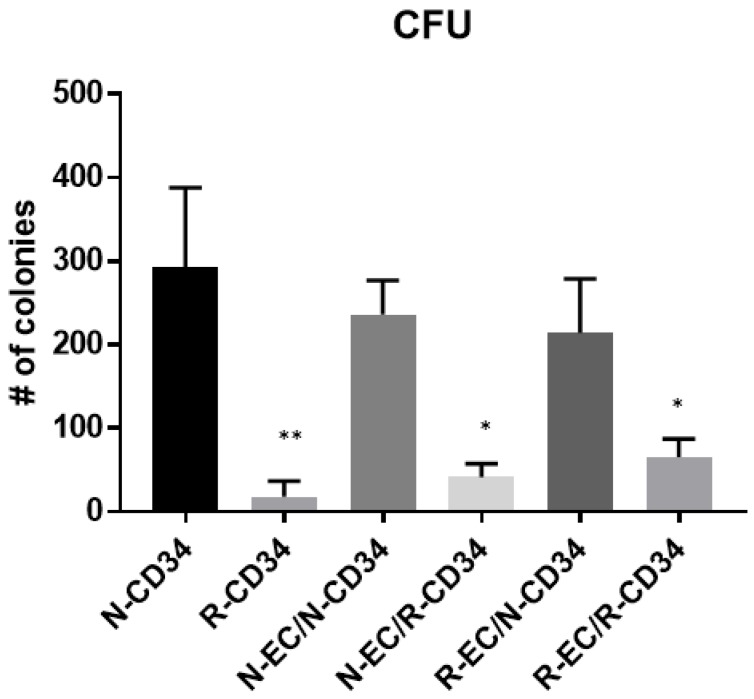
hBMEC slightly improved bone marrow function. Colony formation capability was improved in HSPC cocultured with hBMEC. Significance was determined compared to N-CD34. Error bars represent standard deviation. * *p* < 0.02; ** *p* = 0.006.

The capability of HSPC to form colonies under defined conditions is an indication of differentiation of hematopoietic progenitors. The ability of hBMEC to alter this property was tested using CD34+ HSPC. Nonirradiated (N) HSPC formed colonies, both in the absence and presence of either nonirradiated or irradiated (R) hBMEC (Figure 7). MF radiation (4 Gy) of CD34+ HSPC diminished this capability with statistical significance (*p* = 0.0065). Culture of irradiated CD34+ HSPC with hBMEC for 24 h stimulated recovery of some colonies, although there were still significant differences between these two groups (N-EC/R-CD34 and R-EC/R-CD34) compared to nonirradiated HSPC (*p* = 0.0101; *p* = 0.0162 respectively). 

### 3.6. hBMEC Protected HSPC Phenotype after Radiation

**Figure 8:**
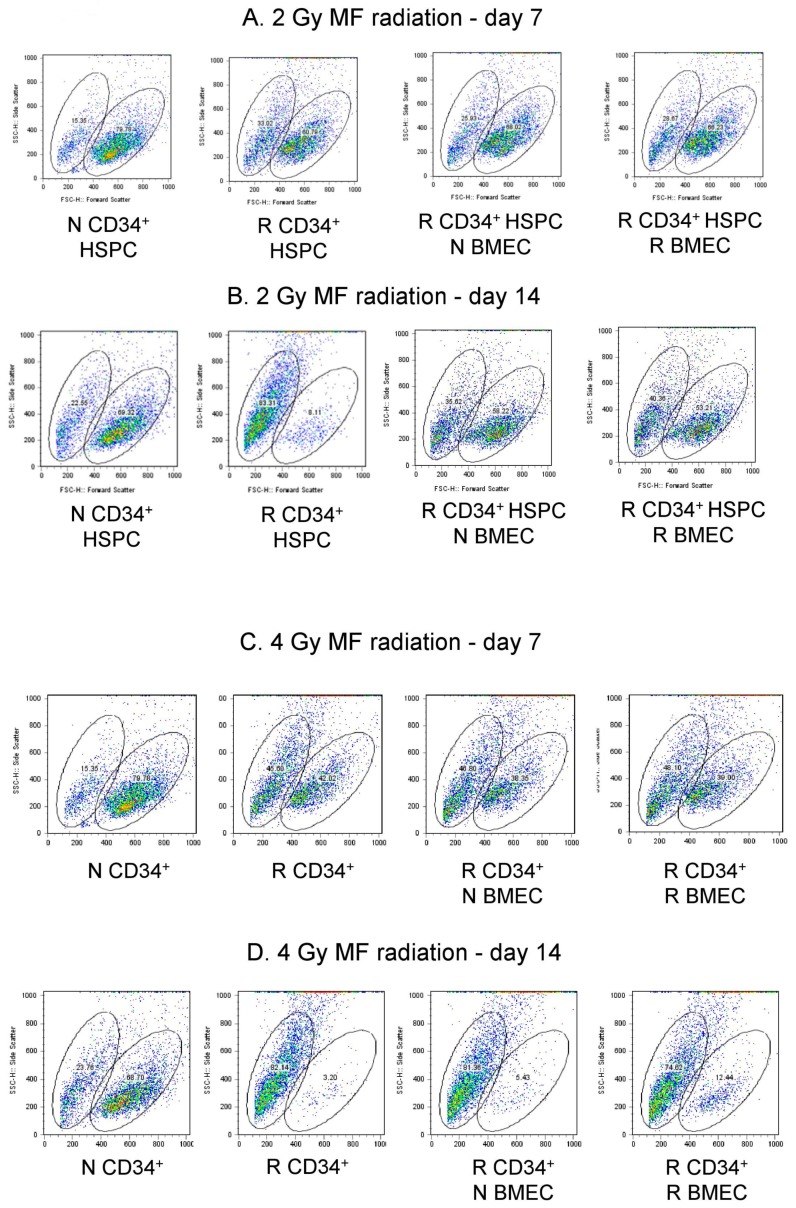
MF radiation and hBMEC altered scatter profile of HSPC. HSPC were irradiated with 2 Gy (**A**,**B**) or 4 Gy (**C**,**D**) for 7 or 14 days. Forward scatter was analyzed by flow cytometry.

**Figure 9:**
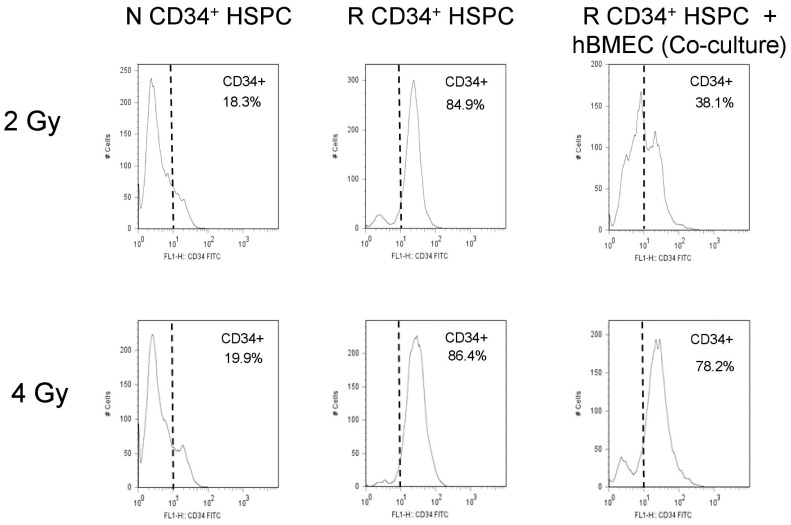
hBMEC altered CD34 expression in HSPC. HSPC were irradiated (2, 4 Gy MF). Cells in coculture were cultured for 24 h with hBMEC postirradiation. All cells were maintained in culture. CD34 expression was analyzed in HSPC by flow cytometry.

Flow cytometry was performed on HSPC seven and 14 days after radiation and 24 h coculture with hBMEC. Scatter profile of nonirradiated HSPC grown for 7 or 14 days in culture reveal two populations of cells, one of larger size than the other based on forward scatter (Figure 8A, Table 1). The larger cells comprised approximately 69–79% of the total cells, and the smaller cells comprised approximately 15–22%. Administration of 2 Gy MF radiation altered the ratio of large to small cell population (5:1 N versus 1.8:1 R) 7 days after culture (79.78% large and 15.35% small in N vs 60.79% large and 33% small cells in R). This trend was not influenced by coculture with hBMEC, either nonirradiated or irradiated (2 Gy MF). HSPC cultured for 14 days after 2 Gy MF radiation exhibited a more pronounced alteration in the ratio of large to smaller sized cells (3.1:1 N versus 0.13:1 R), but coculture with hBMEC protected a percentage of the larger sized cells (Figure 8B, Table 1). The effect of radiation on the ratio of these two cell populations was also observed after 4 Gy MF radiation. hBMEC coculture did not protect the larger cells at this dose, although a slight increase in the larger sized cells was observed on day 14 when R CD34+ were cocultured with R hBMEC (Figure 8C,D, Table 2). On day 14, nonirradiated HSPC in culture stained dimly for the CD34 marker by flow cytometry, while irradiated HSPC in culture stained brighter for CD34. Cells irradiated with 2 Gy MF and cocultured with hBMEC contained both CD34 dim and CD34 bright cells, suggesting that coculture protected a portion of CD34 dim cells, or blocked the transition of cells from a CD34 dim to CD34 bright phenotype (Figure 9). This was a dose-dependent affect; HSPC irradiated at a higher dose (4 Gy) were not protected by coculture with hBMEC. Therefore, interaction of HBMEC with HSPC has direct implications on HSPC differentiation after injury.

## 4. Discussion

The identification of processes involved in immune cell protection, mobilization, and regulation after insult is an area of intense research. In the context of radiation injury, immune cell depletion and recovery have been studied at length, and it is clear in both animal models and in the clinic that the ability to mobilize SPC is essential to radioprotection. Agents such as the CXCR4 chemokine receptor antagonist plerixafor (AMD3100), statins, erythropoietin, vascular endothelial growth factor (VEGF), and angiopoietin-1 have all been shown to effectively promote the peripheral mobilization of CD34+ cells [24,35,36,37]. Neupogen^®^ (filgrastim), the first medical countermeasure currently approved by the FDA for the treatment of radiation induced myelosuppression, acts by stimulating hematopoietic progenitor cell proliferation and differentiation [38,39].

The effect of high LET neutrons on hBMEC has not been established. High LET radiation, because of its densely ionizing nature, creates, among other things, complex DNA damage that is more difficult to repair than that caused by low LET radiation [40]. This was seen in both peripheral blood lymphocytes and hematopoietic progenitor cells [41], and the damage is seen both within the nucleus and the cytoplasm [42]. Our data focus on additional radiation-induced responses, and provide evidence that hBMEC respond to MF radiation via Akt signaling, secretion of cytokines, and changes in protein levels of PARP-1 as well as metalloproteinases MMP1 and MMP9. Also, morphological alterations were seen, which included cell enlargement with increased vesicles and tubular structures, which may be consistent with endothelial cell injury. Morphological changes, including deep invaginations of the luminal surface, large coated vesicles, and tubular structures, were described as indicators of endothelial activation in response to traumatic brain injury [43]. A detailed assessment of endothelial function would be required to determine if hBMEC morphology correlated with activation status in our system.

Alterations in gene expression were analyzed to identify those that were radioresponsive in our model. Although there were genes whose expression was downregulated, the focus for these studies was genes whose expression levels were upregulated two-fold or more. There were five genes encoding angiotensinogen, arichadonate-5-lipoxygenase, Von Willebrand Factor (VWF), IL7, and matrix metalloproteinase 9 (MMP9), whose expression was increased 15-fold or greater after MF radiation. Angiotensinogen, Von Willebrand Factor, MMP9, and Arachidonate-5-lipoxygenase are genes whose protein products are expressed in or involved with EC-mediated functions, including vascular remodeling, hemostasis, and adhesion. Increased VWF protein expression has been reported in EC after gamma-radiation and is associated with endothelial dysfunction [44], and lung and heart pathophysiology [45,46]. Interestingly, decreased VWF was associated with decreased pulmonary fibrosis and increased bone marrow hematopoiesis [47]. Our data do not support this finding, since we saw increased VWF expression in hBMEC and improvement in hematopoiesis from irradiated CD34+ HSPC cultured with hBMEC. Possible reasons for the disparity may be that the studies by Rhieu et al. were performed using mouse total bone marrow versus our study which used purified CD34+ HSPC and human BMEC. MMP9 plays a major role in the degradation of the extracellular matrix (ECM) in a broad range of physiology and pathophysiology processes that involve tissue remodeling, and is upregulated during inflammation [48]. IL7 is a bone marrow derived cytokine produced by nonhematopoietic cells, including lymphatic EC [49] and bone marrow stromal cells [50]. There is a relatively low concentration of IL7 under normal physiological conditions, but under lymphopenic conditions and disease, there is an increase in IL7 transcription from lymphatic EC, as well as increased circulating IL7 [51,52]. Exposure to ionizing radiation at specific dose and radiation qualities can lead to lymphopenia, and the genes for IL7, along with IL10 and Flt3 ligand, encode positive regulators of the lymphoid lineage after TBI [53]. Studies performed using genetically engineered mice showed an increase in IL7 in response to both low dose and high dose gamma-radiation [54]. Although the correlation between radiation exposure and IL7 production is tenuous, we propose that increased IL7 levels in vivo may contribute to relief against radiation induced lymphopenia.

There were multiple genes whose expression in hBMEC was increased approximately 4-fold in response to MF radiation. These include proapoptotic genes (Fas, TNF) and the anti-apoptotic gene BCL2A1 [55]. TNF may induce apoptosis or may activate endothelium, which is critical in inflammation, and can result in an increase in surface expression of selectins and intracellular adhesion molecules (ICAM), leading to an environment that will allow enhanced leukocyte adhesion [56]. Although we detected no change in ICAMs, there is an increase in L-selectin gene expression. Additional endothelial specific genes upregulated include endothelin-2, and the endothelin receptor Type A, both of whose gene expression was increased at least 4-fold. Endothelin-2 message is transiently upregulated in response to low dose radiation and may be a useful biomarker for low-dose irradiation of endothelial tissues [57]. Some genes detected in these arrays were confirmatory of genes upregulated in response to radiation and inflammation, including NOS2, FGF1 and IL6 [58,59]. IL6 is induced in endothelial cells, is within the proinflammatory network, and is associated with senescence-associated secretory phenotypes (SASP) [60].

Global gene expression of hBMEC and HSPC in response to MF radiation displayed unique profiles. IR at a dose of 4 Gy (67% neutron/33% gamma-photons) altered expression of 222 genes in hBMEC, and 2452 genes in HSPC. These data indicate less radioresponsiveness in hBMEC compared to HSPC and the uniqueness of the cell specific genes whose expression levels are altered by radiation. There are 60 genes whose expression was altered in both cell types by both 2 Gy and 4 Gy doses of radiation, and those genes have been categorized into pathways. The largest increases in gene expression occurred in pathways corresponding to cytokines and inflammatory response, and senescence and autophagy; all are consistent with a radiation response and may be potential targets for radiation countermeasures. Genes within pathways associated with Epstein Barr virus latent membrane protein (EBV LMP1) and thymic stromal lymphoprotein (TSLP) suggest that NF-κB signaling through both canonical and noncanonical pathways may occur in these cell types [61,62]. TSLP signaling is upregulated in response to UV radiation and involves hypoxia inducing factor 1 (HIF1) [63]. These signaling pathways, associated with hypoxia, inflammation and radiation response represent areas for further study.

BMEC are in close proximity with HSPC in niches, and the interactions between the two cell types within the niche were also evaluated in the current study. We and others show that BMEC support the proliferation of HSPC in vitro [44,64], and that this specific EC function is not affected by radiation exposure. In vitro coculture systems provide a valuable model for studying cellular communication. The impact of cellular interactions between umbilical cord blood (UCB) hematopoietic cells and BM-derived mesenchymal stem cells (MSCs) included expansion and differentiation of UCB CD34+ cells [65]. We have cocultured HSPC with well characterized hBMEC in our studies [66]. Coculture of nonirradiated and MF-irradiated (4 Gy, 6 Gy) hBMEC with CD34+ HSPC support proliferation, above that seen with HSPC alone. Our studies extend to irradiated HSPC, which due to their radiosensitivity show no proliferation after IR exposure with or without hBMEC support. Direct cell count using trypan blue exclusion suggest that the presence of hBMEC could not protect HSPC from radiation induced cell death (data not shown). Although coculture did not improve proliferation, it caused cellular changes identified by flow cytometry. Alterations in the scatter profile and reduced CD34+ expression of HSPC are characteristic of in vitro differentiation of these cells. MF radiation reduced the number of differentiated CD34- cells, while sparing CD34+ HSPC. This is consistent with the observation that HSPC are less prone to apoptosis than lymphocytes despite similar radiation induced DNA damage [67]. The presence of EC in culture with HSPC rescued a population of larger CD34- cells, providing evidence that supporting niche cells play a role in the radioresponse. These cells may be involved in maintenance of BM activity after radiation, based on increased colonies seen in CFU assays using irradiated HSPC cultured with EC. Further characterization of these cells is necessary to determine the lineage of the rescued cells. It is interesting to note that the radiation status of EC has no bearing on CFU activity, which is an important observation when identifying potential mechanisms of protection. Based on our data, EC function in both direct and indirect ways to protect the viability and function of BM cells against IR. Radiation using neutrons as well as gamma-photons broadens the applicability of these findings, and understanding the interplay between cell types is valuable for the identification of effective radiation treatments. It will be interesting to characterize the size-based subpopulations of HSPC revealed here in terms of molecular and functional phenotypes. The identified genes modulated by radiation and coculture provide guidance for future experiments to test hypotheses concerning specific factors mediating the beneficial effects of BMEC on HSPC. This information will prove useful in the search for medical countermeasures to radiation-induced hematopoietic injury.

## 5. Materials and Methods

### 5.1. Cell Culture

Human BMEC (hBMEC) used in all experiments were established by Dr. Graca Almeida-Porada (Wake Forest School of Medicine; Winston-Salem, NC) [66]. CD34+ HSPC were purchased from Lonza, Inc. (Walkersville, MD). hBMEC were grown on 0.2% gelatin-coated (Biocoat) flasks (Thermo Fisher Scientific Inc. Rockford, IL, USA) in endothelial growth media (Lonza Inc.Walkersville, MD USA) supplemented with 10% fetal bovine serum (FBS). hBMEC were used at ≥75% confluency. HSPC were maintained in HPGM (Lonza, Inc.) supplemented with IL-3, stem cell factor, thrombopoietin, and Flt-3 ligand(HSPC media) (Peprotech, Inc. Rocky Hill, NJ, USA). 

### 5.2. Coculture

**Figure 10:**
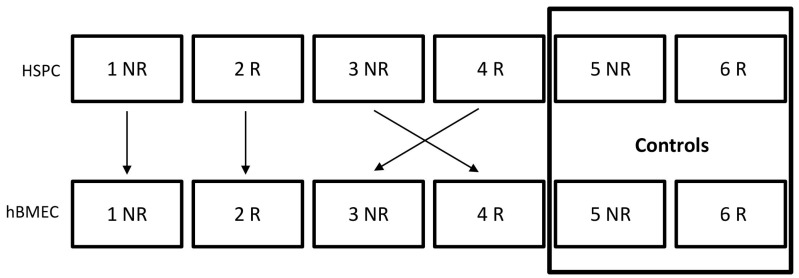
Coculture Strategy. R = irradiated; NR = nonirradiated.

HBMEC and HSPC were irradiated separately. One hour after radiation, HSPC from all conditions were counted, collected by centrifugation, and resuspended in fresh HSPC media. 2–3 × 10^5^ HSPC were added to hBMEC, or to new flasks as controls. Cells were cocultured for 4 or 24 h prior to subsequent experimentation. HSPC are suspension cells and were collected from the culture media for analysis. Coculture combinations are depicted in Figure 10.

### 5.3. MF Irradiation

Cells were irradiated in 25 cm^2^ flasks in the AFRRI TRIGA Irradiation Facility. Two, four, or six Gy total doses were administered at a dose rate of 0.6 Gy/min at ambient temperatures using a standardized technique. The neutron/gamma ratio was approximately 2/1. Variation within the exposure field was less than 4%. Neutron and gamma-photon doses were determined separately using the paired-ionization-chamber technique. The principle of the method is described in the ICRU Report 26 [68] and AAPM Report 7 [69]. Details of its specific implementation at AFRRI are described in the report by Goodman [70]. 

### 5.4. Western Blot

Cells were lysed with RIPA lysis buffer (Thermo Fisher Scientific Inc.) supplemented with protease and phosphatase inhibitor cocktails, and equal amounts of protein were subjected to SDS-PAGE on a 4–12% Tris-Glycine gel using the Mini-Protein Tetra Cell (Bio-Rad Laboratories, Hercules, CA, USA). Proteins were transferred onto a nitrocellulose membrane and probed with antibodies specific for phospho-Akt, Akt, PARP-1, and actin (Cell Signaling Technology, Inc., Danvers, MA, USA). Proteins were detected with appropriate secondary antibodies and ECL detection reagent (GE Healthcare, Pittsburgh, PA, USA).

### 5.5. Protein Array

Secreted proteins were detected in cell culture supernatant using the human cytokine array (Raybiotech, Inc., Norcross, GA, USA) and following the manufacturer’s instructions.

### 5.6. PCR Array Analysis

Changes in gene expression were identified by real-time RT-PCR using the RT^2^ Profiler^TM^ Human Endothelial Cell Biology PCR Array (SABiosciences Corp. Frederick, MD, USA). Briefly, RNA was isolated from cells using RNEasy (Qiagen Sciences Inc. Valencia CA, USA). RNA sample quality was verified using a Nanodrop ND-1000 Spectrophotometer (Thermo Fisher Scientific) and stored at −80 °C until use. RNA was converted into cDNA using the RT^2^ First Strand Kit and the cDNA was used to determine relative gene expression following the manufacturer’s protocol.

### 5.7. Microarray Analysis

Microarray profiling/hybridization was performed at GeneLogic, Inc. (Gaithersburg, MD) using the company’s standard procedures. As described earlier, hBMEC and human CD34+ stem and progenitor cells (HSPC) were irradiated with mixed neutron/gamma radiation at doses of 2 Gy and 4 Gy. RNA was isolated from hBMEC and HSPC 4 h after exposure using a commercially available kit (RNEasy) (Qiagen Sciences Inc.). RNA sample quality was verified using a Nanodrop ND-1000 Spectrophotometer (Thermo Fisher Scientific Inc.) and an Agilent 2100 Bioanalyzer (Agilent Technologies, Santa Clara, CA, USA), with resulting A260/A280 ratios within a range of 1.95 to 2.03 and RNA Integration Number (RIN) ranging from 9.2 to 10. Labeling reactions for the RNA samples were performed using the Quick Amp Two-Color Labeling Kit. The experimental cDNA was labeled with Cy5 (red) and the reference cDNA was labeled with Cy3 (green). The samples were fragmented and each hybridization mixture was loaded onto Agilent Human 4X44K V2 Whole Genome Microarray. The slide was hybridized in an Agilent hybridization chamber at 65 °C with 10 rpm rotation for 17 h, followed by washing per the Agilent protocol. Once dry, the slides were scanned with the Agilent Scanner (G2565BA) using Scanner Version C and Scan Control software version A.8.5.1. Data extraction and quality assessment of the microarray data were completed using Agilent Feature Extraction Software Version 11.0.1.1.

Raw DNA microarray data were background corrected and normalized using an empirical Bayes method as described in Linear Models for Microarray Data (LIMMA) [71,72]. Average log2 expression values (A) and log2 expression ratios (M) were extracted for the following contrasts: MF irradiated BMEC (2 Gy and 4 Gy) versus nonirradiated control and MF irradiated HSPC (2 Gy and 4 Gy) versus nonirradiated control. Genes were ranked based on log expression ratio (M) for each contrast described above. Probe IDs were mapped to gene symbols and Entrez gene IDs using mygene software from R statistical package [73]. Probe IDs that did not map to known genes were filtered out. Then, differentially expressed genes were chosen for each contrast where the absolute log expression ratio > 1 (which is equivalent to a log fold change cutoff of 2). Genes that were commonly or specifically modulated under the experimental conditions were visualized using Venny software package [74]. The 60 genes that were perturbed under all experimental conditions were mapped to pathways. The top eight modulated biological processes and principle component analysis were determined.

### 5.8. Survival and Proliferation

CD34+ HSPC and hBMEC were irradiated with 4 Gy MF radiation independently. 5 × 10^5^ CD34+ HSPC were added to approximately 1 × 10^6^ hBMEC in direct coculture and incubated overnight. CD34+ HSPC were removed from coculture and plated at a density of 5 × 10^4^ cells/mL in triplicate in complete HSPC media. Cell survival and proliferation were determined by direct cell count with trypan blue exclusion using a hemocytometer. Thirty–300 cells were counted for each sample.

### 5.9. Hematopoietic Progenitor Clonogenic Assay

HSPC (5 × 10^3^) were plated onto MethoCult media (StemCell Technologies, Inc., Vancouver, BC), and colony forming units (CFU) were identified and quantified following the manufacturer’s instructions. Colonies were counted 14 days after plating using a Nikon TS100F microscope. Fifty or more cells were considered one colony.

### 5.10. Flow Cytometry

HSPC were stained with anti-CD34 (BD Biosciences, San Jose, CA, USA) and examined by flow cytometry using a FACScalibur (BD Biosciences). Percentages of CD34+ HSPC were analyzed using FlowJo software (Tree Star, Inc. Ashland, OR, USA). 

## Figures and Tables

**Table 1:** CD34^+^ hSPC cell population percentages after 2 Gy MF radiation.

2 Gy	N CD34^+^ HSPC	R CD34^+^ HSPC	R CD34^+^ HSPC + N BMEC	R CD34^+^ HSPC + R BMEC
Scatter Population	Small Large	Small Large	Small Large	Small Large
Day 7	15.35 79.78	33.02 60.79	25.93 68.02	28.67 66.23
Day 14	22.55 69.22	63.31 8.11	35.02 58.22	40.36 53.21

**Table 2:** CD34^+^ hSPC cell population percentages after 4 Gy MF radiation.

4 Gy	N CD34^+^ HSPC	R CD34^+^ HSPC	R CD34^+^ HSPC + N BMEC	R CD34^+^ HSPC + R BMEC
Scatter Population	Small Large	Small Large	Small Large	Small Large
Day 7	15.35 79.78	45.60 42.02	46.80 38.35	48.10 39.00
Day 14	23.76 68.70	62.14 3.20	81.36 5.43	74.82 12.44

## Supplementary Materials

Supplementary materials can be found at https://www.mdpi.com/1422-0067/20/7/1795/s1.

## Abbreviations

ARS	acute radiation syndrome
BM	bone marrow
CFU	colony forming unit
EC	endothelial cell
ECM	extracellular matrix
FBS	fetal bovine serum
HSC/HSPC	hematopoietic stem/hematopoietic stem and progenitor cells
ICAM	intracellular adhesion molecule
IND	improvised nuclear device
IR	ionizing radiation
LET	linear energy transfer
MF	mixed field
MM	multiple myeloma
NR	nonirradiated
R	irradiated
RIN	RNA Integration Number
SASP	senescence-associated secretory phenotypes
SC	stem cell
SCF	stem cell factor
TBI	total body irradiation
ARS	acute radiation syndrome
BM	bone marrow

## References

[1] Jacobson L.O., Simmons E.L., Marks E.K., Robson M.J., Bethard W.F., Gaston E.O. (1950). The role of the spleen in radiation injury and recovery. J. Lab. Clin. Med..

[2] Lorenz E., Uphoff D., Reid T.R., Shelton E. (1951). Modification of irradiation injury in mice and guinea pigs by bone marrow injections. J. Natl. Cancer Inst..

[3] Inoue T., Hirabayashi Y., Mitsui H., Sasaki H., Cronkite E.P., Bullis J.E., Bond V.P., Yoshida K. (1995). Survival of spleen colony-forming units (CFU-S) of irradiated bone marrow cells in mice: Evidence for the existence of a radioresistant subfraction. Exp. Hematol..

[4] Van Vekkum D.W. (1991). Radiation sensitivity of the hemopoietic stem cell. Radiat. Res..

[5] Rodgerson D.O., Reidenberg B.E., Harris A.G., Pecora A.L. (2012). Potential for a pluripotent adult stem cell treatment for acute radiation sickness. World J. Exp. Med..

[6] Pinzur L., Akyuez L., Levdansky L., Blumenfeld M., Volinsky E., Aberman Z., Reinke P., Ofir R., Volk H.D., Gorodetsky R. (2018). Rescue from lethal acute radiation syndrome (ARS) with severe weight loss by secretome of intramuscularly injected human placental stromal cells. J. Cachexia Sarcopenia Muscle.

[7] Giralt S., Costa L., Schriber J., Dipersio J., Maziarz R., McCarty J., Shaughnessy P., Snyder E., Bensinger W., Copelan E. (2014). Optimizing autologous stem cell mobilization strategies to improve patient outcomes: Consensus guidelines and recommendations. Biol. Blood Marrow Transplant..

[8] Arora S., Majhail N.S., Liu H. (2018). Hematopoietic progenitor cell mobilization for autologous stem cell transplantation in multiple myeloma in contemporary era. Clin. Lymphoma Myeloma Leuk..

[9] Juric M., Ghimire S., Ogonek J., Weissinger E., Holler E., Van Rood J., Oudshoorn M., Dickinson A., Greinix H. (2016). Milestones of Hematopoietic Stem Cell Transplantation—From First Human Studies to Current Developments. Front. Immunol..

[10] Singh V.K., Fatanmi O.O., Singh P.K., Whitnall M.H. (2012). Role of radiation-induced granulocyte colony-stimulating factor in recovery from whole body gamma-irradiation. Cytokine.

[11] Bonig H., Papayannopoulou T. (2012). Mobilization of hematopoietic stem/progenitor cells: General principles and molecular mechanisms. Methods Mol. Biol..

[12] Shao L., Luo Y., Zhou D. (2014). Hematopoietic stem cell injury induced by ionizing radiation. Antioxid. Redox Signal..

[13] Ray S., Kulkarni S., Chakraborty K., Pessu R., Hauer-Jensen M., Kumar K., Ghosh S. (2013). Mobilization of progenitor cells into peripheral blood by gamma-tocotrienol: A promising radiation countermeasure. Int. Immunopharmacol..

[14] Singh V., Brown D., Kao T. (2010). Alpha-tocopherol succinate protects mice from gamma-radiation by induction of granulocyte-colony stimulating factor. Int. J. Radiat. Biol..

[15] https://www.fda.gov/emergencypreparedness/counterterrorism/medicalcountermeasures/aboutmcmi/ucm443245.htm.

[16] Zhang J., Niu C., Ye L., Huang H., He X., Tong W., Ross J., Haug J., Johnson T., Fend J. (2003). Identification of the haematopoietic stem cell niche and control of the niche size. Nature.

[17] Xie Y., Yin T., Wiegraebe W., He X., Miller D., Stark D., Perko K., Alexander R., Schwartz J., Grindley J. (2009). Detection of functional haematopoietic stem cell niche using real-time imaging. Nature.

[18] Mikkola H.K., Orkin S.H. (2006). The journey of developing hematopoietic stem cells. Development.

[19] Chotinantakul K., Leeanansaksiri W. (2012). Hematopoietic Stem Cell Development, Niches, and Signaling Pathways. Bone Marrow Res..

[20] Richter R., Forssmann W., Henschler R. (2017). Current Developments in Mobilization of Hematopoietic Stem and Progenitor Cells and Their Interaction with Niches in Bone Marrow. Transfus. Med. Hemother..

[21] Kiel M., Radice G., Morrison J. (2007). Lack of evidence that hematopoietic stem cells depend on N-cadherin-mediated adhesion to osteoblasts for their maintenance. Cell Stem Cell.

[22] Kiel M., Yilmaz O., Iwashita T., Terhorst C., Morrison S. (2005). SLAM family receptors distinguish hematopoietic stem and progenitor cells and reveal endothelial niches for stem cells. Cell.

[23] Luo X., Andres M., Timiryasova T., Fodor I., Slater J., Gridley D. (2005). Radiation-enhanced endostatin gene expression and effects of combination treatment. Technol. Cancer Res. Treat..

[24] Vasa M., Fichtlscherer S., Adler K., Aicher A., Martin H., Zeiher A., Dimmeler S. (2001). Increase in circulating endothelial progenitor cells by statin therapy in patients with stable coronary artery disease. Circulation.

[25] Méndez-Ferrer S., Michurina T.V., Ferraro F., Mazloom A.R., Macarthur B.D., Lira S.A., Scadden D.T., Ma’ayan A., Enikolopov G.N., Frenette P.S. (2010). Mesenchymal and haematopoietic stem cells form a unique bone marrow niche. Nature.

[26] Muramoto G.G., Chen B., Cui X., Chao N.J., Chute J.P. (2006). Vascular endothelial cells produce soluble factors that mediate the recovery of human hematopoietic stem cells after radiation injury. Biol. Blood Marrow Transplant..

[27] Himburg H.A., Muramoto G.G., Daher P., Meadows S.K., Russell J.L., Doan P., Chi J.T., Salter A.B., Lento W.E., Reya T. (2010). Pleiotrophin regulates the expansion and regeneration of hematopoietic stem cells. Nat. Med..

[28] Doan P.L., Himburg H.A., Helms K., Russell J.L., Fixsen E., Quarmyne M., Harris J.R., Deoliviera D., Sullivan J.M., Chao N.J. (2013). Epidermal growthfactor regulates hematopoietic regeneration after radiation injury. Nat. Med..

[29] Yazlovitskaya E., Linkous A., Thotala D., Cuneo K., Hallahan D. (2008). Cytosolic phospholipase A2 regulates viability of irradiated vascular endothelium. Cell Death Differ..

[30] Edwards E., Geng L., Tan J., Onishko H., Donnelly E., Hallahan D. (2002). Phosphatidylinositol 3-Kinase/Akt Signaling in the response of vascular endothelium to ionizing radiation. Cancer Res..

[31] Chou C.H., Chen S.U., Cheng J.C. (2009). Radiation-induced interleukin-6 expression through MAPK/p38/NF-kappaB signaling pathway and the resultant antiapoptotic effect on endothelial cells through Mcl-1 expression with sIL6-Ralpha. Int. J. Radiat. Oncol. Biol. Phys..

[32] Nalla A., Gogineni V., Gupta R., Dinh D., Rao J. (2011). Suppression of uPA and uPAR blocks radiation-induced MCP-1 mediated recruitment of endothelial cells in meningioma. Cell. Signal..

[33] Nirmala C., Jasti S., Sawaya R., Kyritsis A., Konduri S., Ali-Osman F., Rao J., Mohanam S. (2000). Effects of radiation on the levels of MMP-2, MMP-9 and TIMP-1 during morphogenic glial-endothelial cell interactions. Int. J. Cancer.

[34] Clevers H. (2005). Stem cells, asymmetric division and cancer. Nat. Genet..

[35] Liles W., Broxmeyer H., Rodger E., Wood B., Hubel K., Cooper S., Hangoc G., Bridger G., Henson G., Calandra G. (2003). Mobilization of hematopoietic progenitor cells in healthy volunteers by AMD3100, a CXCR4 antagonist. Blood.

[36] Dimmeler S., Aicher A., Vasa M., Mildner-Rihm C., Adler K., Tiemann M., Rütten H., Fichtlscherer S., Martin H., Zeiher A.M. (2001). HMG-CoA reductase inhibitors (statins) increase endothelial progenitor cells via the PI 3-kinase/Akt pathway. J. Clin. Investig..

[37] Llevadot J., Murasawa S., Kureishi Y., Uchida S., Masuda H., Kawamoto A., Walsh K., Isner J., Asahara T. (2001). HMG-CoA reductase inhibitor mobilizes bone marrow—derived endothelial progenitor cells. J. Clin. Investig..

[38] Singh V., Newman V., Seed T. (2015). Colony-stimulating factors for the treatment of the hematopoietic component of the acute radiation syndrome (H-ARS): A review. Cytokine.

[39] Farese A., MacVittie T. (2015). Filgrastim for the treatment of hematopoietic acute radiation syndrome. Drugs Tooday.

[40] Durante M., Loeffler J. (2010). Charged particles in radiation oncology. Nat. Rev. Clin. Oncol..

[41] Rall M., Kraft D., Volcic M., Cucu A., Nasonova E., Taucher-Scholz G., Bönig H., Wiesmüller L., Fournier C. (2015). Impact of charged particle exposure on homologous DNA double-strand break repair in human blood-derived cells. Front. Oncol..

[42] Zhang B., Davidson M., Hei T. (2014). Mitochondria regulate DNA damage and genomic instability induced by high LET radiation. Life Sci. Space Res..

[43] Castejón O. (2013). Increased vesicular and vacuolar transendothelial transport in traumatic human brain oedema. A review. Folia Neuropathol..

[44] Gaugler M., Squiban C., Claraz M., Schweitzer K., Weksler B., Gourmelon P., Van der Meeren A. (1998). Characterization of the response of human bone marrow endothelial cells to in vitro irradiation. Br. J. Haematol..

[45] Kalash R., Berhane H., Au J., Rhieu B., Epperly M., Goff J., Dixon T., Wang H., Zhang X., Franicola D. (2014). Differences in irradiated lung gene transcription between fibrosis-prone C57BL/6NHsd and fibrosis-resistant C3H/HeNHsd mice. In Vivo.

[46] Gabriels K., Hoving S., Seemann I., Visser N., Gijbels M., Pol J., Daemen M., Stewart F., Heeneman S. (2012). Local heart irradiation of ApoE(−/−) mice induces microvascular and endocardial damage and accelerates coronary atherosclerosis. Radiother. Oncol..

[47] Rhieu B., Epperly M., Cao S., Franicola D., Shields D., Goff J., Wang H., Greenberger J. (2014). Increased hematopoiesis in long-term bone marrow cultures and reduced irradiation-induced pulmonary fibrosis in Von Willebrand factor homologous deletion recombinant mice. In Vivo.

[48] Yabluchanskiy A., Ma Y., Iyer R., Hall M., Lindsey M. (2013). Matrix metalloproteinase-9: many shades of function in cardiovascular disease. Physiology.

[49] Iolyeva M., Aebischer D., Proulx S., Willrodt A., Ecoiffier T., Häner S., Bouchaud G., Krieg C., Onder L., Ludewig B. (2013). Interleukin-7 is produced by afferent lymphatic vessels and supports lymphatic drainage. Blood.

[50] Mazzucchelli R., Warming S., Lawrence S., Ishii M., Abshari M., Washington A., Feigenbaum L., Warner A., Sims D., Li W. (2009). Visualization and identification of IL-7 producing cells in reporter mice. PLoS ONE.

[51] Miller C., Hartigan-O’Connor D., Lee M., Laidlaw G., Cornelissen I., Matloubian M., Coughlin S., McDonald D., McCune J. (2013). IL-7 production in murine lymphatic endothelial cells and induction in the setting of peripheral lymphopenia. Int. Immunol..

[52] Gao J., Zhao L., Wan Y., Zhu B. (2015). Mechanism of action of IL-7 and its potential applications and limitations in cancer immunotherapy. Int. J. Mol. Sci..

[53] Tsuboi I., Hirabayashi Y., Harada T., Hiramoto M., Kanno J., Inoue T., Aizawa S. (2008). Predominant regeneration of B-cell lineage, instead of myeloid lineage, of the bone marrow after 1 Gy whole-body irradiation in mice: Role of differential cytokine expression between B-cell stimulation by IL10, Flt3 ligand and IL7 and myeloid suppression by GM-CSF and SCF. Radiat. Res..

[54] Shin S., Lee K., Kang Y., Kim K., Lim S., Yang K., Kim J., Nam S., Kim H. (2011). Differential expression of immune-associated cancer regulatory genes in low-versus high-dose-rate irradiated AKR/J mice. Genomics.

[55] Werner A., de Vries E., Tait S., Bontjer I., Borst J. (2002). Bcl-2 family member Bfl-1/A1 sequesters truncated bid to inhibit is collaboration with pro-apoptotic Bak or Bax. J. Biol. Chem..

[56] Collins T., Read M., Neish A., Whitley M., Thanos D., Maniatis T. (1995). Transcriptional regulation of endothelial cell adhesion molecules: NF-kappa B and cytokine-inducible enhancers. FASEB J..

[57] Lanza V., Fadda P., Iannone C., Negri R. (2007). Low-dose ionizing radiation stimulates transcription and production of endothelin by human vein endothelial cells. Radiat. Res..

[58] Gorbunov N., Pogue-Geile K., Epperly M., Bigbee W., Draviam R., Day B., Wald N., Watkins S., Greenberger J. (2000). Activation of the nitric oxide synthase 2 pathway in the response of bone marrow stromal cells to high doses of ionizing radiation. Radiat. Res..

[59] Hong C., Kim Y., Pyo H., Lee J., Kim S., Lee S., Noh J. (2013). Involvement of inducible nitric oxide synthase in radiation-induced vascular endothelial damage. J. Radiat. Res..

[60] Coppé J., Desprez P., Krtolica A., Campisi J. (2010). The senescence-associated secretory phenotype: the dark side of tumor suppression. Annu. Rev. Pathol..

[61] Lavorgn A., Harhaj E. (2012). EBV LMP1: New and shared pathways to NF-κB activation. PNAS.

[62] Gewurz B.E., Towfic F., Mar J.C., Shinners N.P., Takasaki K., Zhao B., Cahir-McFarland E.D., Quackenbush J., Xavier R.J., Kieff E. (2012). Genome-wide siRNA screen for mediators of NF-κB activation. PNAS.

[63] Jang Y., Jeoing S., Park Y., Bae H., Lee H., Ryu W., Park G., Son S. (2013). UVB Induces HIF-1-dependent TSLP expression via the JNK and ERK pathways. J. Investig. Dermatol..

[64] Wang Q., Yan Q. (2013). Soluble factors from bone marrow endothelial cells regulate differentiation and proliferation of hematopoietic and endothelial lineages and embryonic stem cells. Acta Physiol..

[65] Da Silva C., Gonçalves R., Dos Santos F., Andrade P., Almeida-Porada G., Cabral J. (2010). Dynamic cell-cell interactions between cord blood haematopoietic progenitors and the cellular niche are essential for the expansion of CD34(+), CD34(+)CD38(−) and early lymphoid CD7(+) cells. J. Tissue Eng. Regen. Med..

[66] Almeida-Porada G., Ascensão J. (1996). Isolation, characterization, and biologic features of bone marrow endothelial cells. J. Lab. Clin. Med..

[67] Durdik M., Kosik P., Kruzliakova J., Jakl L., Markova E., Belyaev I. (2017). Hematopoietic stem/progenitor cells are less prone to undergo apoptosis than lymphocytes despite similar DNA damage response. Oncotarget.

[68] (1977). Neutron Dosimetry for Biology and Medicine.

[69] (1980). Protocol for Neutron Beam Dosimetry.

[70] (1985). A Practical Guide to Ionization Chamber Dosimetry at the AFRRI Reactor.

[71] Smyth G. (2004). Linear models and empirical bayes methods for assessing differential expression in microarray experiments. Stat. Appl. Genet. Mol. Biol..

[72] Ritchie M., Phipson B., Wu D., Hu Y., Law C., Shi W., Smyth G. (2015). Limma powers differential expression analyses for RNA-sequencing and microarray studies. Nucleic Acids Res..

[73] Chunlei W., Mark A., Su A. (2014). MyGene.info: Gene Annotation Query as a Service. http://biorxiv.org/content/biorxiv/early/2014/09/17/009332.full.pdf.

[74] Oliveros J. (2007). VENNY. An Interactive Tool for Comparing Lists with Venn Diagrams. http://bioinfogp.cnb.csic.es/tools/venny/index.html2github.com/benfred/venn.js.

